# Prediction of Conventional Oxygen Therapy Failure in COVID-19 Patients With Acute Respiratory Failure by Assessing Serum Lactate Concentration, PaO2/FiO2 Ratio, and Body Temperature

**DOI:** 10.7759/cureus.21987

**Published:** 2022-02-07

**Authors:** Simon E Fridman, Pasquale Di Giampietro, Annamaria Sensoli, Michelle Beleffi, Cristina Bucce, Veronica Salvatore, Fabrizio Giostra, Alice Gianstefani

**Affiliations:** 1 Emergency Department, University Hospital of Bologna Sant’Orsola-Malpighi Polyclinic (IRCCS Azienda Ospedaliero-Universitaria di Bologna - Policlinico di Sant'Orsola-Malpighi), Bologna, ITA

**Keywords:** prognostic tool, lactate, emergency department, respiratory failure, covid, lot score

## Abstract

One of the challenges that emerged during the coronavirus disease 2019 (COVID-19) pandemic and is still relevant today is the need to identify patients with acute respiratory failure (ARF) who could benefit from conventional oxygen therapy (COT) - oxygen supplementation with nasal cannulas, Venturi masks, and non-rebreather masks - without recurring to advanced respiratory therapy, such as high-flow nasal cannula (HFNC), continuous positive airway pressure (CPAP), non-invasive ventilation (NIV), or invasive mechanical ventilation. The aim of the study was to develop a clinical tool able to predict the failure of COT in COVID-19 patients presenting to the emergency department (ED) with ARF. This was a retrospective monocentric cohort study carried out in the ED of the University Hospital of Bologna Sant’Orsola-Malpighi Polyclinic, Italy. The cohort comprised 101 COVID-19 patients with ARF from the first pandemic wave who received COT. This cohort was used to develop a scale that considers serum lactate concentration, partial arterial oxygen pressure/inspired oxygen fraction (PaO_2_/FiO_2_) ratio, and body temperature to predict COT failure, referred to as the Lactate, Oxygenation, and Temperature (LOT) score. The highest possible score was 17 points. The LOT score was associated with COT failure (area under the receiver operating curve or AUROC = 0.79, 95% CI 0.69 - 0.89, *p* < 0.001); the cut-off value of > 5 points had optimal predictive power and showed significantly higher 30-day mortality (log-rank χ^2^ = 28,828, *p* < 0.0001). The LOT score was able to effectively predict COT failure in COVID-19 patients with ARF. Patients with LOT score > 5 had a very high risk of therapy failure, and more advanced respiratory therapies must be considered in these patients.

## Introduction

In December 2019, a cluster of patients with severe pneumonia of unknown origin was identified in Wuhan, China [1]. The disease rapidly spread and evolved into a global pandemic [2]. The causing agent was soon identified as severe acute respiratory syndrome coronavirus 2 (SARS-CoV-2) and the disease it caused was named coronavirus disease 2019 (COVID-19) [3].

The spectrum of the clinical manifestations of COVID-19 ranges from asymptomatic to critical disease, leading to multiorgan failure and death [4-5]. At admission to the emergency department (ED), patients who present with acute respiratory failure (ARF) can be classified into different phenotypes, requiring different management approaches and therapeutic strategies [6-7]. Some patients may not require oxygen therapy at all while others may already present a severe acute respiratory distress syndrome (ARDS) and require immediate intubation and intensive care. COVID-19 patients with ARF, but whose clinical condition is not critical at the moment of admission to the ED, present the clinician with a challenge: to correctly and precisely estimate the risk of disease and ARF progression and to choose the optimal treatment, specifically the ideal oxygen or respiratory therapy. This task is often further complicated by the availability of resources, such as hospital beds, ventilators, and healthcare professionals, which may change from one local reality to another and from one moment to another.

There are many methods for administering supplemental oxygen (SO): with or without ventilatory support, invasive and non-invasive. COT delivers SO non-invasively and without providing any ventilatory support, making use of nasal cannulas (NCs), simple face masks (SFMs), Venturi masks (VMs), and non-rebreather masks (NRBs). High-flow nasal cannulas (HFNCs) are also used to deliver SO, but have many advantages over the simpler methods [8] and may provide low levels of positive end-expiratory pressure (PEEP), although inconsistently. Continuous positive airway pressure (CPAP) devices and more advanced options, such as non-invasive ventilation (NIV) and invasive mechanical ventilation (IMV), all provide a consistent PEEP [9], with the exception of pure pressure support ventilation (PSV) without PEEP, which is rarely used.

Much research has been done in the field of ARF management in COVID-19 patients, and guidelines were established to aid in critical clinical decisions [10-13]. Patients without ARDS but in need of SO have received much less attention [14]. The ROX (Respiratory rate - OXygenation) index, proposed by Roca et al. [15] for the prediction of HFNC failure in patients with pneumonia and ARF, was shown to correlate with the need for hospital admission, mechanical ventilation, and mortality risk in COVID-19 patients [16]. The HACOR score (Heart rate, Acidosis, Consciousness level, Oxygenation and Respiratory rate), proposed by Duan et al. [17] for the prediction of NIV failure in hypoxemia due to several causes, was evaluated for the prediction of CPAP failure in COVID-19 patients with ARF [18] and was found to be comparable in its predictive power to the partial arterial oxygen pressure/inspired oxygen fraction (PaO_2_/FiO_2_ or P/F) ratio.

Many experts believe that SARS-CoV-2 will not be eradicated and that the COVID-19 clinical manifestations will become less severe over time [19-22]. In this study, we aimed to evaluate the efficacy of COT in non-severe COVID-19 patients presenting to the ED with ARF by developing a clinical scoring system for the prediction of its failure.

This article was previously presented at the 2021 edition of the Area Critica Congress in Bologna, Italy, in December 2021.

## Materials and methods

Study design

We conducted a retrospective monocentric cohort study in the ED of the University Hospital of Bologna Sant’Orsola-Malpighi Polyclinic, a 1500-bed tertiary care teaching hospital in Northern Italy with approximately 70,000 yearly ED attendances. COVID-19 patients presenting to the ED with ARF during the months of March and April 2020, the first COVID-19 pandemic wave in Italy, who received COT (NCs, VMs, NRBs), were enrolled in the study, excluding patients who did not have ARF at presentation or severe patients who started any form of advanced respiratory therapy (HFNC, CPAP, NIV, or IMV) at admission; patients not admitted to the hospital were also excluded from the study. The diagnosis of COVID-19 was based on a positive polymerase chain reaction (PCR) test for SARS-CoV-2 on a nasopharyngeal swab performed at either admission to the ED or during hospitalization.

Clinical charts and hospital electronic records were used as data sources. The data were recorded by the ED attending physicians as part of their routine patient care. We then extracted the relevant data points from the clinical records and compiled them into a separate database. Exposure variables were assessed at hospital admission and included: patient demographics, medical history, symptoms, vital signs, and arterial blood gas (ABG) analysis. End-point variables were assessed from admission to discharge or demise of the patient and included: serial ABGs during the first 48 hours from admission to the ED, oxygen and respiratory therapies used during hospitalization, and in-hospital mortality.

The main outcome analyzed was the failure of COT determined by the fulfillment of at least one of the following conditions: (i) refractory ARF defined as persistence of P/F ratio < 150 after 48 hours of COT; (ii) worsening of ARF defined as a reduction in the P/F ratio after 48 hours of COT to < 300 or such that would constitute a progression from one ARDS severity sub-class to another as per the Berlin definition of ARDS [23]; (iii) escalation to any form of advanced respiratory therapy (HFNC, CPAP, NIV, or IMV); (iv) death during hospitalization. In addition, we registered the date of hospital discharge and post-discharge all-cause mortality. The last follow-up date is 14/04/2021.

Statistical analysis

Data were analyzed using the statistical software SPSS 26 (IBM Corp., Armonk, NY). Data are reported as mean and standard deviation (SD). We used the non-parametric Mann-Whitney U test for all continuous variables while categorical variables were analyzed using the chi-squared test. The ability to predict the failure of PEEP-less oxygenation was determined using the area under the receiver operating curve (AUROC). A p-value of < 0.05 was considered to be statistically significant.

We developed the risk model as follows. First, we used univariate analysis to identify variables associated with the trial failure. Second, variables with a p-value < 0.1 in the univariate analysis were included in a stepwise multivariate logistic regression analysis to identify independent risk factors associated with COT failure. The absence of collinearity was ensured by calculating the Spearman’s ρ coefficients. The probability of stepwise was 0.05 for entry and 0.1 for removal. We then obtained a regression model. We evaluated the final model for goodness-of-fit using the Homer-Lemeshow test (p > 0.05). Third, we used the method suggested by Sullivan et al. to create the clinical score [24]. We classified the variables in the final model into clinically meaningful categories and recorded the midpoint value in each category. For each variable, we set a category with the lowest risk for failure as the within-group reference and assigned it zero points; we then calculated the weight in each category multiplying the β regression coefficient by the difference between the category midpoint value and the within-group reference value. Finally, we assigned 1 point to the category with the lowest weight overall and set this weight as the between-groups reference. To assign points to the other categories, we divided the weight of each unassigned category by the between-groups reference value and rounded off the result to the nearest integer value. The score is determined by the sum of the points for each variable.

Survival probability at 30 days and 120 days from admission to the ED based on the same cut-off value used for the prediction of COT failure was analyzed with Kaplan-Meier survival curves.

## Results

Between March 11, 2020, and April 27, 2020, there were a total of 101 admissions to the ED of COVID-19 patients meeting our criteria. The mean age of participants was 73.2 ± 15.9 years, and 47% were female. The study cohort descriptive statistics are reported in Table 1.

**Table 1:** Study cohort descriptive statistics COPD: chronic obstructive pulmonary disease, TIA: transient ischemic attack, GCS: Glasgow Coma Scale, BP: blood pressure, MAP: mean arterial pressure, SpO2: peripheral oxygen saturation, ABG: arterial blood gas, Δ (A-a): O2 alveolar-arterial oxygen gradient

Variable	All patients (n = 101)		Variable	All patients (n = 101)
Age, years, mean ± SD	73.2 ± 15.9		Fatigue	11 (10.9)
Gender, n (%)			Myalgias or arthralgias	10 (9.9)
Male	54 (53.5)		Diarrhea	7 (6.9)
Female	47 (46.5)		Dysosmia/Dysgeusia	2 (2)
Comorbidities, n (%)			GCS < 15	21 (20.8)
Hypertension	59 (58.4)		Vital signs, mean ± SD	
Diabetes mellitus	13 (11.9)		Systolic BP, mmHg	121.5 ± 19.3
Smoking	8 (7.9)		Diastolic BP, mmHg	71.1 ± 11.6
COPD	25 (24.8)		MAP, mmHg	87.9 ± 12.6
Asthma	3 (3)		Heart rate, beats/min	88 ± 17.7
Other respiratory diseases	8 (7.9)		Respiratory rate, breaths/min	23.6 ± 7
Ischemic heart disease (IHD)	15 (14.9)		SpO_2_, %	92.5 ± 4.8
Oncologic condition	8 (7.9)		Body temperature, °C	37.5 ± 0.9
Chronic kidney disease (CKD)	11 (10.9)		ABG analysis, mean ± SD	
History of stroke or TIA	12 (11.9)		pH	7.5 ± 0.1
Immunodeficiency	2 (2)		PaCO_2_, mmHg	32 ± 6
Clinical features, n (%)			PaO_2_, mmHg	56.9 ± 10.2
Fever	93 (92.1)		HCO_3_^-^, mmol/L	23.6 ± 3.3
Shortness of breath (SOB)	63 (62.4)		Lactate, mmol/L	1.3 ± 0.6
Cough	51 (50.5)		P/F ratio	267.6 ± 50
Sore throat	3 (3)		Δ (A-a) O_2_	53.5 ± 11.9
Headache	2 (2)		ROX index	20.3 ± 7.1

The most frequent comorbidities were hypertension and chronic obstructive pulmonary disease (COPD). More than half reported both shortness of breath (SOB, 62%) and coughing (51%). The majority of patients (79%) were neurologically intact with a Glasgow coma scale (GCS) of 15. Mean blood pressure (BP) and heart rate (HR) values were within the normal reference ranges (BP 121.5 ± 19.3 / 71.1 ± 11.6 mmHg, HR 88 ± 17.7 beats/min), while the respiratory rate (RR) and peripheral oxygen saturation (SpO_2_) were altered in a big portion of patients (RR 23.6 ± 7 breaths/min, SpO_2_ 92.5 ± 4.8 %). Almost all patients were febrile (92%) with a mean body temperature (BT) of 37.5 ± 0.9 °C.

Arterial blood gas analysis performed in room air (FiO_2_ 21%) frequently documented respiratory alkalosis (PaCO_2_ 32 ± 6 mmHg) with partial renal compensation (HCO_3_^-^ 23.6 ± 3.3) and a mean pH value of 7.5 ± 0.1. Blood oxygenation was largely inadequate with a PaO_2_ mean value of 56.9 ± 10.2 and a mean P/F ratio of 267.6 ± 50; the alveolar-arterial oxygen gradient (Δ (A-a) O_2_) recorded in most patients was higher than expected for age with a mean value of 53.5 ± 11.9. None of the patients had lactic acidosis (Lac 1.3 ± 0.6 mmol/L). The ROX index in the study cohort was 20.3 ± 7.1.

Statistically significant differences were observed between the group of patients in which COT succeeded and failed, as shown in Table 2. In the univariate analysis, we included only variables for which sufficient data samples were available. The respiratory dynamics and gas exchange were worse in the failed group: RRs were higher while SpO_2_, PaO_2,_ and consequently, the P/F ratios were lower, resulting in higher values of Δ (A-a) O_2_ and lower ROX indices. BTs and serum lactate concentrations were also higher in the failed group. Mean HR was higher in the failed group, but the difference was not statistically significant (p = 0.064). The HACOR score was very low and practically the same in both groups (1.1 ± 2.7 vs. 0.9 ± 2.0).

**Table 2:** Baseline characteristics of the cohort based on outcome (COT failure) COPD: chronic obstructive pulmonary disease, GCS: Glasgow coma scale, BP: blood pressure, MAP: mean arterial pressure, SpO2: peripheral oxygen saturation, ABG: arterial blood gas, Δ (A-a) O2: alveolar-arterial oxygen gradient

Variable	COT succeeded (n = 36)	COT failed (n = 65)	p-value
Age, years	69.6 ± 16.9	75.1 ± 15.1	0.130
Gender, n (%)			0.603
Male	18 (17.8)	36 (35.6)	
Female	18 (17.8)	29 (28.7)	
Comorbidities, n (%)			
Hypertension	19 (18.8)	40 (39.6)	0.392
COPD	7 (6.9)	18 (17.8)	0.358
Clinical features, n (%)			
Shortness of breath	19 (18.8)	44 (43.6)	0.138
Cough	21 (20.8)	30 (29.7)	0.241
GCS < 15	8 (7.9)	13 (12.9)	0.792
Vital signs, mean ± SD			
Systolic BP, mmHg	121.6 ± 17.2	121.5 ± 20.5	0.829
Diastolic BP, mmHg	73.3 ± 12.1	70 ± 11.3	0.293
MAP, mmHg	89.4 ± 12.4	87.1 ± 12.8	0.456
Heart rate, beats/min	83.6 ± 15.4	90.6 ± 18.5	0.064
Respiratory rate, breaths/min	21.4 ± 6.5	24.8 ± 7	0.023
SpO_2_, %	94.1 ± 4	91.5 ± 5	0.004
Body temperature, °C	37.2 ± 0.8	37.7 ± 1	0.019
ABG analysis, mean ± SD			
pH	7.5 ± 0.1	7.5 ± 0.1	0.525
PaCO_2_, mmHg	33.2 ± 6.6	31.4 ± 5.6	0.168
PaO_2_, mmHg	60.6 ± 8.3	54.8 ± 10.6	0.002
HCO_3_^-^, mmol/L	24.2 ± 2.9	23.3 ± 3.6	0.181
Lactate, mmol/L	1.1 ± 0.5	1.4 ± 0.7	0.004
P/F ratio	288.6 ± 39.6	255.9 ± 51.5	0.001
Δ (A-a) O_2_	48.3 ± 9.3	56.5 ± 12.4	<0.001
ROX index	22.7 ± 8	18.9 ± 6.1	0.009

In the multivariate analysis, we omitted SpO_2_, PaO_2_, Δ (A-a) O_2_ gradient, and ROX index. In general, directly measured variables were preferred to calculated variables. SpO_2_ is easier to measure than PaO_2_, however, the latter is more accurate and considering the other statistically significant variables, it was clear that the ABG test was necessary as a basis for the score derivation. The Δ (A-a) O_2_ gradient resulted to be collinear with both the PaO_2_ and P/F ratio (ρ = -7.3 for both) and was thus omitted. The PaO_2_ and P/F ratio resulted to be, as expected, collinear (ρ = 0.95) and we opted for the P/F. The ROX index and RR were also collinear (ρ = 0.98), and we preferred RR for its simplicity and because it was not dependent on previously omitted variables.

After the multivariate analysis, we ended up with three variables independently associated with COT failure: serum lactate concentration, P/F ratio, and BT. These three variables were used to develop a risk-scoring system to predict COT failure. Following the weights for each variable, we assigned 4 points to BT, 4 points to serum lactate concentration, and 9 points to P/F (Table 3). We named the score LOT (Lactate, Oxygenation, and Temperature), on a scale of a total of 17 points.

**Table 3:** Final model for prediction COT failure (LOT score) β: regression coefficient per unit increase, COT: conventional oxygen therapy, LOT: Lactate, Oxygenation, and Temperature

Variable	β_i_	Category (_j_)	Reference value (W_ij_)	β_i_(W_ij_ - W_i __REF_)	Points
Lactate, mmol/L	10.324				
		< 1.0	0.5 = W_1__ REF_	-	0
		1.0 - 1.4	1.2	0.7227	1
		1.5 - 1.9	1.7	12.389	2
		≥ 2.0	2.5	20.648	4
P/F ratio	-0.0189				
		≥ 400	415 = W_2__ REF_	-	0
		325 - 399	363	0.9828	2
		250 - 324	288	24.003	4
		175 - 249	213	38.178	7
		< 175	150	50.085	9
Body temperature, °C	0.8075				
		< 37.0	36.5 = W_3__ REF_	-	0
		37.0-37.4	37.2	B = 0.5653	1
		37.5-37.9	37.7	0.9690	2
		38.0-38.9	38.5	16.150	3
		≥ 39	39.5	24.225	4

The AUROC for the prediction of failure was 0.79 (0.69 - 0.89), p < 0.001. We identified the cut-off value of 6 points as having optimal predictive power: sensitivity (SE) 77.8%, specificity (SP) 69.7%, positive likelihood ratio (LR+) 2.57, negative likelihood ratio (LR-) 0.32, positive predictive value (PPV) 83.1%, and negative predictive value (NPV) 62.2%.

COT failed in 64.36% of patients of the cohort. The LOT score in the study cohort was 7.72 ± 2.84, and it was ≥ 6 in 69.31% of patients. In patients with LOT score < 6, the failure rate was 13.86%. However, in those with LOT score ≥ 6, the failure rate was 48.52%. Higher LOT scores were associated with increased failure rates.

**Figure 1:**
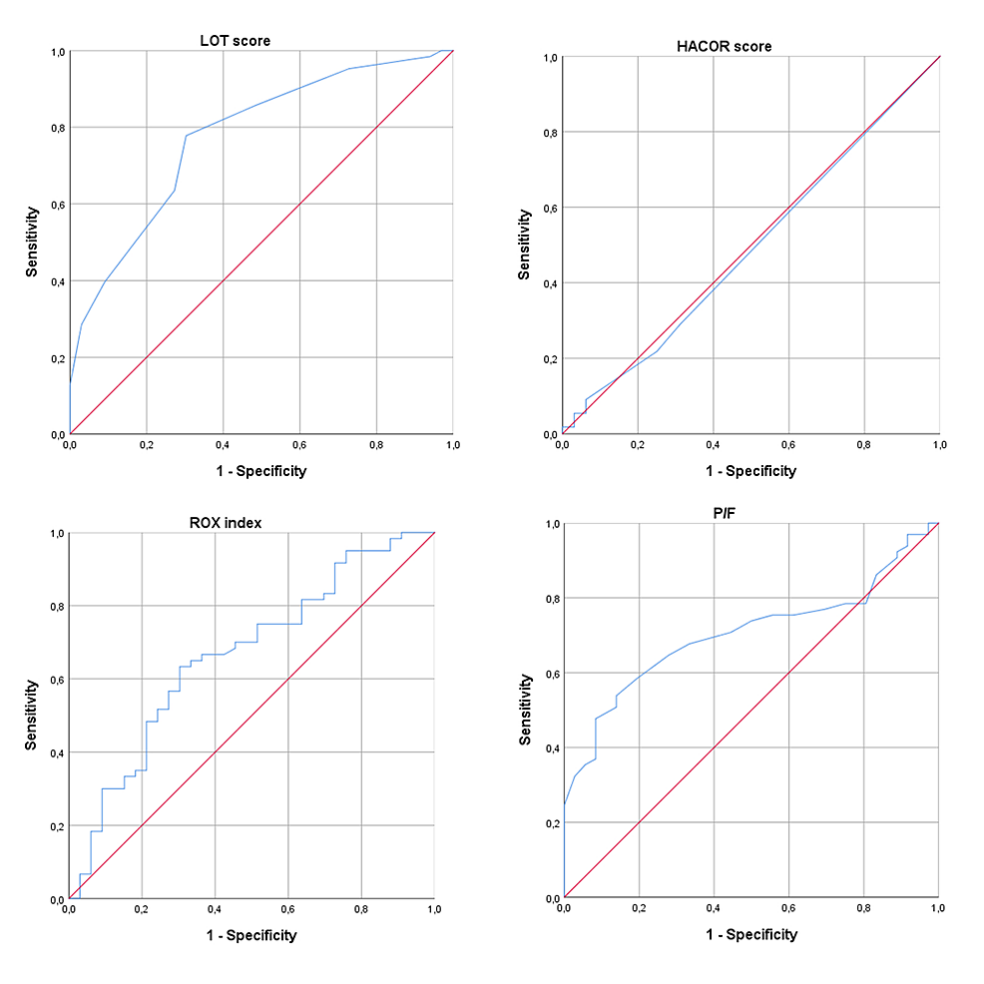
ROCs for different prediction models ROC: receiver operating curve

The LOT score was able to better predict failure of COT relative to the HACOR score, the ROX index, and the P/F ratio alone, as shown in Figure 1 and Table 4.

**Table 4:** AUROCs for the different prediction models AUROC: area under the receiver operating curve, LOT: Lactate, Oxygenation, and Temperature, HACOR: Heart rate, Acidosis, Consciousness level, Oxygenation and Respiratory rate, ROX: Respiratory rate - OXygenation

Prediction model	AUROC (95% CI)	p value
LOT score	0.79 (0.69 – 0.89)	< 0.001
HACOR score	0.49 (0.36 – 0.62)	0.89
ROX index	0.66 (0.55 – 0.78)	0.009
P/F ratio	0.70 (0.60 – 0.80)	0.001

**Figure 2:**
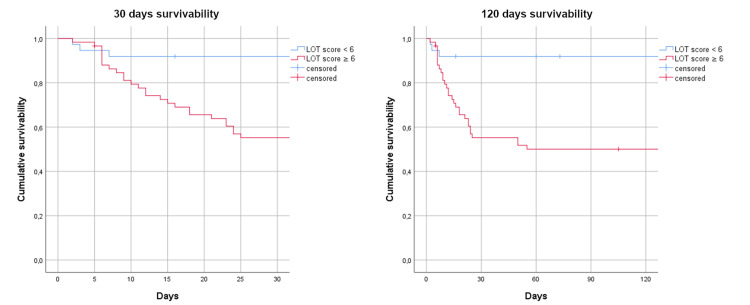
Kaplan-Meier curves for survivability below and the above LOT score cut-off value LOT: Lactate, Oxygenation, and Temperature

We used the same cut-off for the analysis of survivability (Figure 2). Most patients with a LOT score of ≥ 6 died in the first month from admission to the ED. At the four months follow-up, the survivability was comparable. A log-rank comparison between the two groups, below and above the cut-off value, confirmed it was statistically significant (χ2 = 28,828, p < 0.0001).

## Discussion

The LOT score was developed to aid clinicians in identifying and managing COVID-19 patients with ARF in which COT would be applicable and to allow for timely therapy escalation where appropriate. Ideally, this tool could allow for better and more sustainable resource allocation and, more importantly, to avoid potential iatrogenic complications related to hospital stay and ventilation. For example, Gattinoni et al. talked about the need to consider the phenotype when choosing the right therapy in the case of ARF [6]. In patients with the L phenotype (low elastance = high compliance), COT could be effective and may prevent a potential ventilator-induced lung injury (VILI) or patient self-inflicted lung injury (P-SILI).

We compared the performance of the LOT score with other existing scores and clinical parameters. The HACOR score was not able to predict COT failure. Guia et al. observed a significant correlation of HACOR scores > 5 with CPAP failure in COVID-19 patients with ARF [18]. In their study cohort, however, patients seemed to be in more severe clinical conditions, with higher RRs and significantly lower P/F ratios compared to the cohort of patients we evaluated. Indeed, in our population, very few patients had HACOR scores > 5. In addition, the HACOR score was conceived as a tool to predict NIV failure in hypercapnic patients [17,25-26] while most patients with COVID-19 are hypocapnic due to hyperventilation. The ROX index did correlate with COT failure but presented poor predictive power of COT failure relative to the LOT score. The ROX index is based on the S/F ratio (SpO_2_/FiO_2_) rather than the P/F ratio, but FiO_2_ was uninfluential, as it was fixed at 21%, and SpO_2_ was collinear with PaO_2_, which is directly proportional to the P/F, so the contribution of either S/F or P/F ratios to the predictive power or the scales should be comparable. Considering the remaining variables used by these two scales, it is apparent that serum lactate concentration and BT are better predictors of COT failure relative to RR. The P/F ratio, unsurprisingly, correlated well with COT failure, but it was still outperformed by the LOT score and is probably less useful for the prediction of COT failure in patients with higher P/F ratios.

It was interesting to see the correlation between serum lactate levels and COT failure, as practically all patients had normal serum lactate concentrations. It is known that lactate is produced in response to lung injury, most prominently in ARDS [27], but it does not necessarily rise above the normal limits in other types of ARF [28]. It has already been observed that in most COVID-19 patients, serum lactate levels remain within the normal range [29]. It is possible that the normal reference range for serum lactate is not sensitive enough for the upregulation of lung lactate production in COVID-19 pneumonia, but it still seems quite useful to stratify the patients based on their normal yet varying serum lactate levels.

BT is one of those clinical features that may differ greatly from one COVID-19 patient to another. In a big meta-analysis, almost 80% of adult COVID-19 patients presented with fever; the prevalence of low and medium-grade fevers was higher compared to high-grade fever [30]. Another meta-analysis found that fever that is not particularly high might still be associated with severe COVID-19 [31]. The LOT score assigns points to patients with a BT as low as 37°C, and discriminates patients in the low and mid-ranges of higher-than-normal BTs (37°C - 38.9°C), assigning the maximum number of points to patients with BT ≥ 39°C. It is interesting that, yet again, a clinical parameter routinely used but easily underestimated in COVID-19 patients, especially in cases of mild disease, reveals itself as particularly valuable and refocuses our attention to what we would otherwise consider practically normal or at least expected. Fever was also found to be associated with mortality in another study [32].

Although the aim of the study was to predict failure of COT in COVID-19 patients with ARF, we discovered that the LOT score correlated in a very significant manner with 30-days all-cause mortality using the same cut-off identified as optimal for the prediction of COT failure. This is important because a stable patient with a mild disease might have a LOT score of 6 or higher, and it is exactly the type of patient that would be at risk of undertreatment and/or underestimation of his or her clinical condition.

To our knowledge, this is the first study that evaluated the efficacy of COT in COVID-19 patients with ARF. This type of therapy is more tolerable and has fewer iatrogenic complications, is less expensive, more widely available, and does not require an intensive or semi-intensive care unit bed. It is plausible to assume that in patients in whom such therapy is sufficient, more advanced respiratory therapies would result in more harm than benefit.

The cohort of patients studied is part of the first COVID-19 pandemic wave in Italy, which by itself is a limiting factor for two main reasons. First, we now know that patient characteristics changed between pandemic waves [33-34]. Second, at the beginning of the crisis, there was little information on the disease, and clinical practice was largely based on individual professional judgment rather than on policy and evidence-based guidelines or protocols. This resulted in heterogeneous management of COVID-19 patients both in the ED and during hospitalization, and decisions pertaining to therapy escalation or de-escalation and their timing definitely had an impact on this statistical analysis, as they determined the outcome of COT failure.

COVID-19 epidemiology changes not only over time but also from one location to another [35-36]. Continuous virus mutation and variants distribution, population immunization (both natural and artificial), geopolitics, socio-demographics, etc. all contribute to the specific characteristics of the patients in different areas of the world. Therefore, it is likely that the cohort of COVID-19 patients from our center is not universally representative.

BT can and is measured in different ways [37]. There is virtually no standardization with regards to BT measurement in clinical practice, a subject that has been debated for decades, and best practices and guidelines exist for the different instruments and modalities [38]. The COVID-19 pandemic has introduced contactless infrared systems, a technology very rarely used before in clinical practice, which complicated things even more [39]. The retrospective nature of our study makes it impossible to identify the exact method used to measure the temperature and thus measurement may be inaccurate and/or biased. Nevertheless, we believe that being a monocentric study and considering the fact that the professionals who work in our ED adhere to the same general practices and conventions, BT measurements are likely reproducible locally, and if bias was present, it is probably systematic. This means that it is vital that a standard technique and specific equipment be used when measuring BT and that the scoring system may need to be adjusted in the future based on results from other institutes or over time.

The P/F ratio is a highly inaccurate parameter by its nature because it depends on the estimated FiO_2_, which, unfortunately, is very difficult to estimate in many circumstances, as very well summarized by Tobin et al. [40]. Most notably, in cases of hyperventilation and when using NRBs, it is almost always estimated incorrectly. Nonetheless, the P/F value used in the calculation of the LOT score is obtained from an ABG analysis performed in room air at the moment of admission to the ED, so the FiO_2_ is fixed at 21% and the P/F ratio is precise. We also used serial ABG analyses performed during the first 48 hours after admission to determine whether COT had failed. The P/F ratios obtained from these ABG analyses were of course less accurate because the patients were receiving SO with modalities that render FiO_2_ estimation problematic, however, the key factor was the trend observed rather than the single measurements. It would still be better to put in place a clear and standardized method for determining the FiO_2_ with specific oxygen delivery systems, interfaces, and oxygen flow rates. In this case too, future adjustments to the scoring system may be needed.

## Conclusions

We found that the LOT score was able to effectively predict failure of COT in COVID-19 patients with ARF. A higher score indicated higher chances of therapy failure. The score can be readily calculated, as all it takes is a thermometer and a point-of-care blood gas analyzer, both of which are routinely used in the setting of an emergency department. Patients with a LOT score of > 5 had a very high risk of therapy failure. In these high-risk patients, more advanced respiratory therapies must be considered. The LOT score is designed to predict failure, not success, and thus it cannot safely exclude failure in patients with low scores.

We believe that the LOT score, once validated, could be a very useful tool in the hands of clinicians facing a mild-to-moderate COVID-19 patient with ARF and would permit them to quickly and easily identify patients who require more advanced treatments. Additional studies are necessary to validate the use of the LOT score in the ever-evolving context of the disease, possibly with a multicentric prospective randomized design.
